# Vaccinia virus strain NYVAC induces substantially lower and qualitatively different human antibody responses compared with strains Lister and Dryvax

**DOI:** 10.1099/vir.0.2008/004440-0

**Published:** 2008-12

**Authors:** Claire M. Midgley, Mike M. Putz, Jonathan N. Weber, Geoffrey L. Smith

**Affiliations:** 1 Department of Virology, Faculty of Medicine, Imperial College London, Norfolk Place, London W2 1PG, UK; 2 Division of Medicine, Imperial College London, Norfolk Place, London W2 1PG, UK

## Abstract

The antibody responses elicited by immunization of humans with vaccinia virus (VACV) strains Lister, Dryvax and NYVAC have been determined and compared. Neutralizing antibodies against intracellular mature virus (IMV) and extracellular enveloped virus (EEV), and binding antibody titres (ELISA) against the EEV protein B5, the IMV proteins A27 and H3, and VACV-infected cell lysate were measured. Lister and Dryvax induced broadly similar antibody titres, consistent with the fact that these vaccines each protected against smallpox. In contrast, antibody titres induced by NYVAC were significantly lower than those induced by both Lister and Dryvax. Moreover, there were qualitative differences with NYVAC-immunized subjects failing to induce A27-specific antibodies. These observations suggest that although NYVAC is a safer VACV strain, it does not induce an optimal VACV-specific antibody response. However, NYVAC strains engineered to express antigens from other pathogens remain promising candidate vaccines for immunization against other diseases.

Variola virus (VARV), the causative agent of smallpox, was eradicated in 1977 by widespread vaccination with vaccinia virus (VACV) but without an adequate understanding of the protective mechanisms (Fenner *et al.*, 1988). The WHO reference vaccine was a calf lymph preparation of VACV strain Lister, although several other strains were used, including NYCBH (Dryvax), EM-63 and Tian Tan (Fenner *et al.*, 1988). These vaccine preparations had imperfect safety records and, consequently, with current concerns regarding bioterrorism and revaccination (Smith & McFadden, 2002), modern and safer vaccines are needed. With no means of testing the efficacy of new vaccines clinically, comparative studies with conventional vaccine strains are required (Greenberg *et al.*, 2005; Putz *et al.*, 2006; Davies *et al.*, 2008).

During the smallpox eradication campaign, several attenuated VACV strains were produced and tested, including CVI-78 (Rivers, 1931), modified vaccinia Ankara (MVA) (Mayr & Munz, 1964) and LC16m8 (Hashizume *et al.*, 1985), derived from NYCBH, Ankara and Lister, respectively. Although these strains were safer, their efficacy is uncertain because vaccinees were not exposed to smallpox. VACV strain NYVAC was developed as a vaccine vector by deletion of 18 ORFs from VACV strain Copenhagen (Tartaglia *et al.*, 1992). NYVAC also has potential as a safer smallpox vaccine and because it is highly attenuated, while retaining good immunogenicity (Tartaglia *et al.*, 1992). NYVAC protected mice against VACV challenge (Belyakov *et al.*, 2003) and was safe in immunocompromised macaques (Edghill-Smith *et al.*, 2003) although protective longevity is uncertain (Ferrier-Rembert *et al.*, 2008). NYVAC was also used safely in clinical trials (Konishi *et al.*, 1998; Ockenhouse *et al.*, 1998; Bart *et al.*, 2008; Harari *et al.*, 2008; McCormack *et al.*, 2008) but its efficacy against smallpox is unknown.

VACV morphogenesis generates two antigenically distinct infectious forms of virion: the intracellular mature virus (IMV) and the extracellular enveloped virus (EEV) (Smith *et al.*, 2002). IMV is surrounded by one membrane, whereas EEV has a second membrane containing virus antigens absent from IMV. Both IMV- and EEV-specific antibodies (Abs) confer some protection, although EEV-specific Abs are more important (Boulter & Appleyard, 1973; Law *et al.*, 2005), and both are long-lived after immunization/infection (Hammarlund *et al.*, 2003; Putz *et al.*, 2005, 2006). In humans, H3 (Davies *et al.*, 2005; Putz *et al.*, 2006) and A27 are targets of IMV-neutralizing Abs and B5 is the only target of EEV-neutralizing Abs (Bell *et al.*, 2004; Putz *et al.*, 2006). In animal models, immunization with each of these proteins confers some protection (Lai *et al.*, 1991; Hooper *et al.*, 2000, 2003; Fogg *et al.*, 2004; Hooper *et al.*, 2004; Pulford *et al.*, 2004; Davies *et al.*, 2005; Lustig *et al.*, 2005; Heraud *et al.*, 2006; Xiao *et al.*, 2007).

Benchmark Ab titres against several important VACV antigens following immunization with VACV strain Lister were established (Putz *et al.*, 2006). Here, these benchmarks were compared to responses elicited by another licensed smallpox vaccine, Dryvax and the attenuated VACV strain, NYVAC. Vaccines were compared by (i) IMV-specific neutralizing Abs and (ii) ELISA against recombinant EEV (B5) and IMV (A27 and H3) antigens and against VACV-infected cell lysate (VACV). All methods have been described previously (Putz *et al.*, 2006). Neutralizing Ab titres (ND_50_, serum dilution reducing the number of plaques by 50 %) were determined by plaque reduction neutralization (PRN) of sucrose-gradient purified VACV strain Western Reserve (WR) IMV. ELISAs used WR-infected cell lysate and WR recombinant B5 and A27, expressed in mammalian CHO cells and in *Escherichia* *coli*, respectively. Bacterially expressed H3 was a gift from Huw Davies (UC Irvine, USA).

Ab responses following immunization with VACV strains Lister and Dryvax were compared (Fig. 1). Lister serum samples were available from naïve individuals (*n*=13) and revaccinees (*n*=69) at days 0 and 21, 6 months and 1 year post-vaccination (Auckland *et al.*, 2005; Putz *et al.*, 2006). Sera from individuals immunized with Dryvax were from both VACV-naïve participants (*n*=24) and revaccinees (*n*=25) at day 0 and 30 days post-vaccination. Vaccine comparisons at day 0 and ‘1 month’ (days 21 or 30) were made for naïve and revaccinee groups independently by Kruskal–Wallis test with Dunn's multiple comparison (Prism 3.0).

In general, Ab responses were both quantitatively and qualitatively similar between Lister and Dryvax, in both titres (Fig. 1) and targets (Supplementary Fig. S1 available in JGV Online). No differences were observed against the VACV cell lysate (Fig. 1). B5 responses were also similar although, in revaccinees, levels were higher (*P*<0.05) after immunization with Dryvax. Addition of ≤10 μg recombinant H3 and A27 to these sera, reduced IMV neutralization partially, indicating that both antigens induced neutralizing Abs in each vaccine (Supplementary Fig. S1). As reported for Lister-induced responses (Putz *et al.*, 2006), depletion of B5 Abs completely abrogated, or very greatly reduced, the EEV-neutralizing activity of Dryvax sera, indicating that B5 is the only target of EEV-neutralizing Abs in both vaccines (Supplementary Fig. S1).

However, some differences were observed in the kinetics of responses to different antigens (Fig. 1). One month after vaccination, H3 titres were lower in naïve individuals immunized with Lister compared with Dryvax (*P*<0.001). Only 46 % of individuals seroconverted (defined as fourfold increase in titre) against H3 after Lister vaccination, whereas 100 % seroconverted after Dryvax immunization. This is consistent with recombinant H3 protein reducing the neutralizing activity of Dryvax (Supplementary Fig. S1) but not Lister sera. This may reflect strain- or time-dependent differences. Following primary VACV infections, Ab responses peak at about days 28–30 (Frey *et al.*, 2003; Greenberg *et al.*, 2005). So at day 21, when the Lister samples were collected, the H3 Abs may be submaximal, whereas the Dryvax samples were collected nearer the time of peak titres. Similarly, A27 responses were higher in naïve individuals immunized with Dryvax compared with Lister (*P*<0.001). Antigen variation is unlikely to explain these differences because the proteins share 99 to 100 % aa identity between these VACV strains (www.poxvirus.org).

Interestingly, unlike other antigens, the A27 titres were not significantly different in revaccinees compared with naïve individuals after Lister and Dryvax vaccination. Following revaccination, Abs peak at day 14 (Stienlauf *et al.*, 1999; Frey *et al.*, 2003; Greenberg *et al.*, 2005). Possibly in revaccinees, by days 21 or 30, A27 Abs are already declining, suggesting differing kinetics compared with other antigens. Following Dryvax vaccination, IMV-neutralizing Abs followed a similar pattern, with titres being slightly lower, though not significantly, in revaccinees compared with naïve individuals. This was surprising but it was reported previously that, 28 days after immunization, IMV-neutralizing responses were not different between vaccination groups (Kennedy *et al.*, 2004; Greenberg *et al.*, 2005; Treanor *et al.*, 2006). In this study, A27 binding Abs in Dryvax revaccinees correlated strongly with neutralizing Abs (Spearman, *r*=0.8090; *P*<0.0001), more than with other antigens. In contrast, 21 days after immunization with Lister, neutralizing Abs in revaccinees correlated most strongly with VACV binding Abs (Spearman *r*=0.8599, *P*<0.0001) and only moderately with individual antigens (Putz *et al.*, 2006).

Ab responses to Lister and NYVAC were compared next (Fig. 2). Sera were obtained from individuals immunized with recombinant NYVAC encoding HIV-1 (Clade C) *gag*, *pol*, *env*, *nef* [(Bart *et al.*, 2008), EuroVacc 01 trial, www.eurovacc.org]. Naïve participants (*n*=7) and revaccinees (*n*=7) were immunized at day 0 and week 4 and sera were collected at day 0, and at weeks 4, 8, 24 and 48 following vaccination. The day 21 and 4 weeks samples from the Lister and NYVAC trials, respectively, were compared as ‘1 month’. These same day 21 Lister samples were also compared to week 8 NYVAC samples; herein described as ‘2 months’. Naïve and revaccinee groups were compared independently (Kruskal–Wallis test, Dunn's multiple comparison). The 6 months and 24 weeks samples and the 1 year and 48 weeks samples were also compared. At these times only two vaccines were studied, and naïve and revaccinee groups were compared independently (Mann–Whitney *U* test).

The major differences between Lister and NYVAC were that (i) NYVAC induced significantly lower levels of Abs and (ii) NYVAC did not induce any A27-specific Abs. For A27, none of the previously naïve individuals was seropositive even after two NYVAC doses. Some revaccinees were seropositive due to residual Abs from previous smallpox vaccination(s) but no individuals exhibited increased levels of A27-specific Abs. In addition, depletion of A27-specific Abs from participants immunized twice with NYVAC had no effect on IMV neutralization (Supplementary Fig. S1). These observations are consistent with A27 not being expressed in NYVAC-infected HeLa cells (Najera *et al.*, 2006). This difference is probably not due to antigen variation between VACV strains because the A27 proteins from both viruses share 99 % aa identity (www.poxvirus.org).

In general, NYVAC elicited weaker responses than Lister against both EEV (B5-ELISA) and IMV (VACV-ELISA) in all individuals. After one dose of NYVAC, the geometric mean increase in titres were 1.0- to 3.8-fold, whereas increases after Lister vaccination were up to 17.1-fold. In addition, 4 of 14 individuals immunized with NYVAC did not seroconvert to any of the antigens tested and only one individual seroconverted against B5, H3 and VACV. In contrast, 70 of 72 individuals seroconverted after being immunized with Lister. After two doses of NYVAC (2 months), B5 and VACV responses were similar to Lister-induced responses. By 6 and 12 months, however, NYVAC-induced Abs declined to levels below those induced by Lister. In addition, in revaccinees, the second dose of NYVAC did not boost Ab titres. The fold increase between 4 and 8 weeks in this group was only 1- to 1.7-fold, indicating that NYVAC demonstrates very limited boosting efficacy in vaccinated individuals. If a protective Ab level is greater than that induced 1 year after boosting with a licensed vaccine (Lister re-vac) (Putz *et al.*, 2005), then NYVAC would not be deemed protective. These findings are consistent with the observation that in mice, even after two NYVAC immunizations, (i) neutralizing Ab levels were significantly lower 150 days post-immunization compared with Lister-induced levels, and (ii) long-term protection was poorer following NYVAC immunization (Ferrier-Rembert *et al.*, 2008).

H3 responses after NYVAC vaccination were unusual. Responses induced by two doses of NYVAC ≥ those elicited by Lister in revaccinees or naïve individuals, respectively (2 months, *P*<0.05). NYVAC-induced H3 responses were also maintained over 1 year to levels equal to that in revaccinees immunized with Lister. Possibly, this maintenance of H3-specific Abs may be influenced by the lack of A27-specific Abs. NYVAC-induced H3-specific Abs were compared to IMV PRN titres and a strong correlation was found (Spearman, *r*=0.8724, *P*<0.001); much stronger than that for Lister samples. However, incubation of sera with up to 10 μg recombinant H3, only reduced the neutralizing capacity by a maximum of 15 % (Supplementary Fig. S1). This indicates that, even without A27, other IMV-neutralizing targets must be present. This is consistent with a recent study highlighting the flexibility and redundancy in IMV-neutralizing Abs (Benhnia *et al.*, 2008).

Following vaccination with two doses of NYVAC (‘2 month’ samples), IMV-neutralizing Abs were surprisingly low. No significant differences were observed between the VACV ELISA titres induced by NYVAC and Lister but, in revaccinees, NYVAC-induced neutralizing Ab levels were significantly lower than Lister-induced titres (*P*<0.01, Fig. 3a[Fig f3]). As such, even though VACV ELISA titres correlated strongly with IMV PRN titres for both vaccines (Lister, Spearman *r*=0.8529, *P*<0.0001; NYVAC, Spearman *r*=0.7168, *P*=0.0006), the lines of best fit provided different equations (Fig. 3b[Fig f3]). For a given VACV ELISA titre, the neutralizing Ab titre induced by NYVAC would be lower than that induced by Lister. This reflects the lack of an A27 response and/or differences in other neutralizing targets. For example, in addition to A27 neither L1 nor A17 (two targets of IMV-neutralizing Abs) are expressed in NYVAC-infected HeLa cells (Najera *et al.*, 2006). It is also possible that low neutralizing Ab levels are elicited because the infection is replication-deficient and new virions are not produced. This finding demonstrates the importance of using functional assays, such as neutralizing assays, to assess the efficacy of new vaccine strains.

In summary, binding and neutralizing Ab levels following immunization with VACV strains Lister, Dryvax and NYVAC were determined and compared. In general, Dryvax induced quantitatively and qualitatively similar responses to Lister. Some antigen-specific Abs did differ in magnitude but this probably reflects the times of sample collection. The similarities between these two effective vaccines provide some insight into the protective mechanisms involved. In contrast, NYVAC-induced Ab levels were lower than those of Lister, even after two NYVAC vaccinations. Similarly, MVA induced lower neutralizing responses than Dryvax in naïve individuals, even after two MVA vaccinations, but responses were similar in the short-term in revaccinees (Parrino *et al.*, 2007). In addition, no qualitative differences were observed between MVA- and Dryvax-induced Abs against neutralizing targets (Davies *et al.*, 2008). However, important qualitative differences were detected here between NYVAC and Lister. These differences render NYVAC an unlikely new smallpox vaccine. Although safe for patients in whom smallpox vaccination is contraindicated, NYVAC may not elicit strong enough responses to be efficacious.

Our findings also suggest that kinetics of different antigen-specific Abs may vary following infection and that this, in turn, influences neutralizing Ab kinetics. In addition, with different vaccine strains and as the time since vaccination changes, although VACV-specific ELISA titres may correlate with IMV-neutralizing titres, a certain ELISA titre will not necessarily always correspond to a specific IMV-neutralizing titre. These differences highlight the need for independent neutralization analysis and the importance of multiple correlates of immunity.

## Supplementary Material

[Supplementary Figure]

## Figures and Tables

**Fig. 1. f1:**
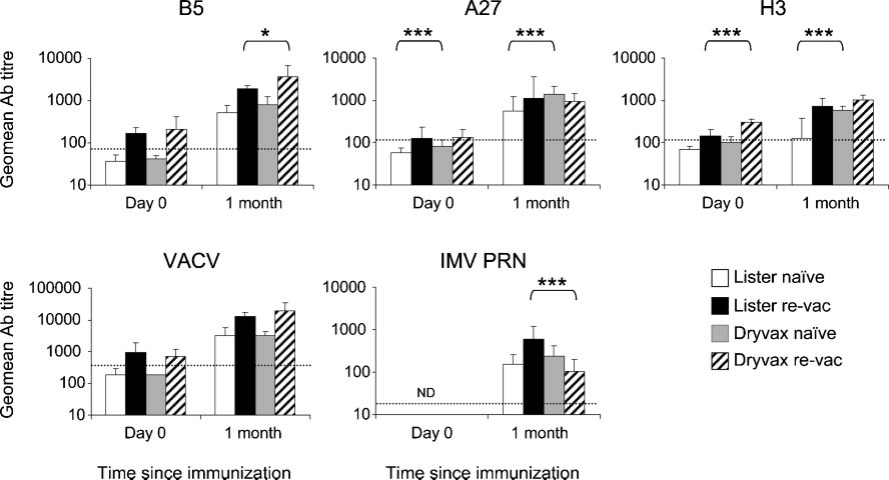
Comparison of Ab responses elicited by Lister and Dryvax in naïve individuals (naïve) and in revaccinees (re-vac). B5-, A27-, H3- and whole virus (VACV)-specific Ab ELISA end-point titres and IMV-specific neutralizing (ND_50_) Ab titres were determined. Cut-off levels for seropositivity for each assay (dotted lines) and statistically significant differences between naïve groups and between re-vac groups (*, *P*<0.05; ***, *P*<0.001) are depicted. Error bars represent 95 % confidence intervals. nd, Not determined.

**Fig. 2. f2:**
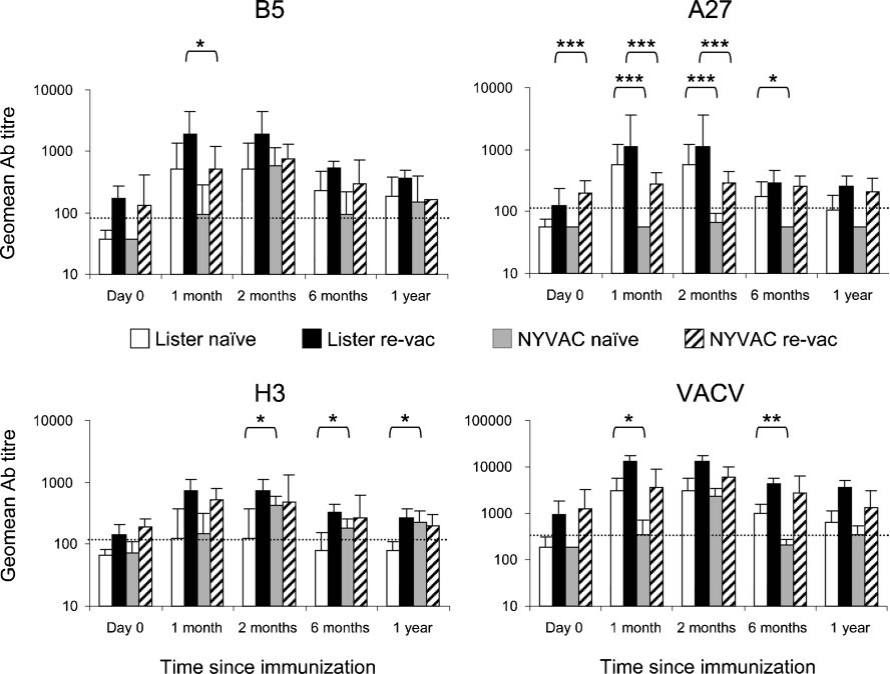
Comparison of binding Ab responses elicited by Lister and NYVAC in naïve individuals (naïve) and in revaccinees (re-vac). Cut-off levels for seropositivity for each assay (dotted lines) and statistically significant differences between naïve groups and between re-vac groups (*, *P*<0.05; **, *P*<0.01; ***, *P*<0.001) are depicted. Error bars represent 95 % confidence intervals.

**Fig. 3. f3:**
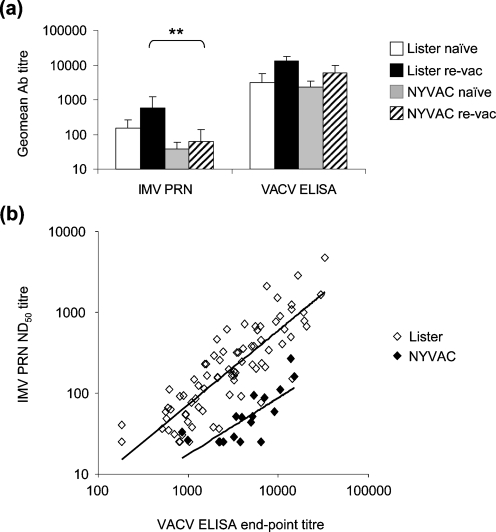
Comparison of binding and neutralizing Ab responses elicited by Lister and NYVAC. (a) VACV-specific binding (VACV ELISA) Ab levels and IMV-specific neutralizing (IMV PRN) Ab levels detected at the ‘2 month’ time point after immunization with Lister or NYVAC are compared. Responses in naïve individuals (naïve) and in revaccinees (re-vac) are shown. Statistically significant differences between naïve groups and between re-vac groups (**, *P*<0.01) are depicted. Error bars represent 95 % confidence intervals. (b) Correlations between VACV-specific binding Ab titres and IMV-specific neutralizing Ab titres for individuals immunized with Lister or NYVAC. Lines of best fit are depicted.
