# Supplementary material for: Vaccinia virus strain NYVAC induces substantially lower and qualitatively different human antibody responses compared with strains Lister and Dryvax
Source: J Gen Virol. 2008 Dec;89(Pt 12):2992–7. doi: 10.1099/vir.0.2008/004440-0 (PMC2885029; doi:10.1099/vir.0.2008/004440-0)
Supplement: [Supplementary Figure] [file supp_89_12_2992__index.html]

 Vaccinia virus strain NYVAC induces substantially lower and qualitatively different human antibody responses compared with strains Lister and Dryvax -- Midgley et al. 89 (12): 2992 Data Supplement - Supplementary Figure -- Journal of General Virology

## 

### Vaccinia virus strain NYVAC induces substantially lower and qualitatively different human antibody responses compared with strains Lister and Dryvax, by C. M. Midgley, M. M. Putz, J. N. Weber and G. L. Smith

*Journal of General Virology* vol. **89**, part 12, pp. 2992 - 2997

**Supplementary Fig. S1.** Neutralizing activity of human sera after antibody depletion  [PDF]  (26 KB)

  
  
