# Supplementary material for: Vaccinia virus strain NYVAC induces substantially lower and qualitatively different human antibody responses compared with strains Lister and Dryvax
Source: J Gen Virol. 2008 Dec;89(Pt 12):2992–7. doi: 10.1099/vir.0.2008/004440-0 (PMC2885029; doi:10.1099/vir.0.2008/004440-0)
Supplement: [Supplementary Figure] [file supp_89_12_2992__1.pdf]

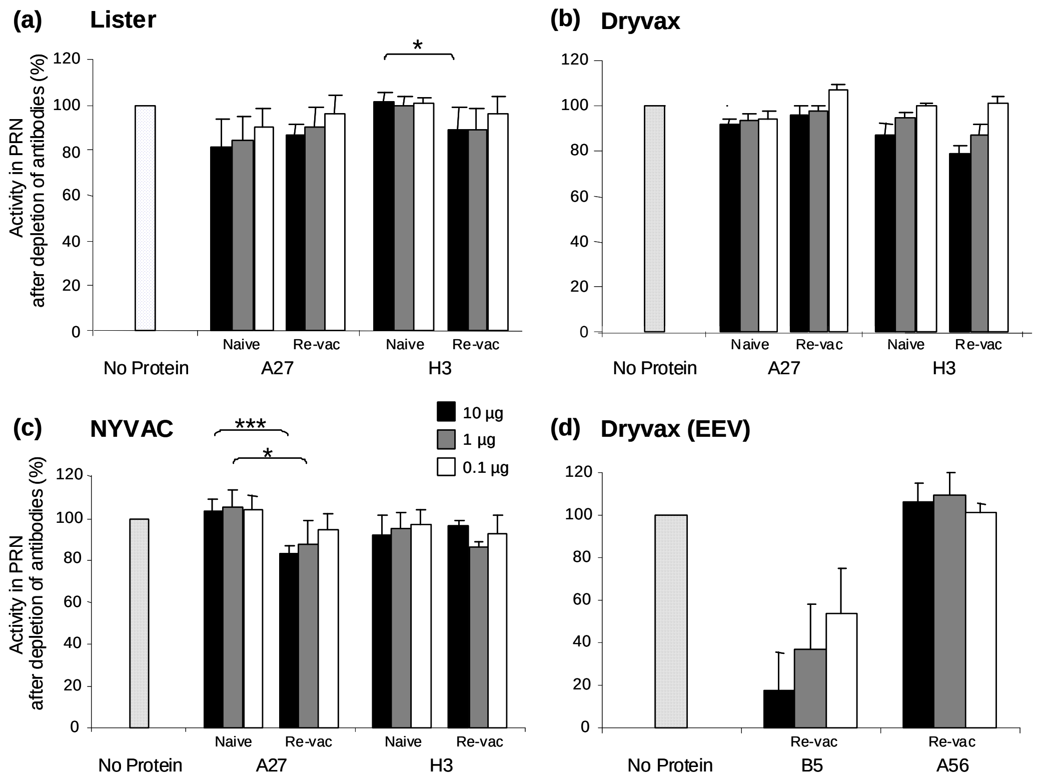

Supplementary Fig. S1. Neutralizing activity of human sera after antibody depletion. Sera from individuals immunized with (a) Lister (naïve, *n*=6; re-vac, *n*=6), (b) Dryvax (naïve, *n*=6; re-vac, *n*=6) and (c) NYVAC (naïve, *n*=5; re-vac, *n*=5) collected 21 days post-vaccination, were incubated with 0.1, 1 or 10 µg of purified recombinant A27 or H3 for 1 h at 37 °C, to deplete A27- or H3-specific Abs, respectively. The remaining IMV-neutralizing activity was determined in duplicate and compared to untreated serum. (d) Sera from individuals immunized with Dryvax (*n*=6, all revaccinees) were incubated with 0.1, 1 or 10 µg of purified recombinant B5 or A56 for 1 h at 37 °C, to deplete B5- or A56-specific Abs. A standard EEV PRN was then performed and the remaining EEV-neutralizing activity was determined and compared to untreated serum. In each case, a concentration of serum corresponding to one ND_{50} titre was used. As a positive control for the assay, sera were incubated with up to 4×10^7 p.f.u. of UV-inactivated sucrose-gradient purified IMV (data not shown; Putz *et al.*, 2006). Statistically significant differences between naïve and revaccinees are indicated (*, $P<0.05$; ***, $P<0.001$; Student's *t*-test). Error bars indicate the 95% confidence intervals.

Reference

Putz, M. M., Midgley, C. M., Law, M. & Smith, G. L. (2006). Quantification of antibody responses against multiple antigens of the two infectious forms of Vaccinia virus provides a benchmark for smallpox vaccination. *Nat Med* **12**, 1310–1315. Medline

---

Midgley, C. M., Putz, M. M., Weber, J. N. and Smith, G. L. (2008). Vaccinia virus strain NYVAC induces substantially lower and qualitatively different human antibody responses compared with strains Lister and Dryvax. *J Gen Virol* **89**, 2992–2997.
